# A Multitask Time–Frequency Deep Learning Approach for Anesthesia Depth Monitoring and Transition Prediction

**DOI:** 10.3390/diagnostics16121937

**Published:** 2026-06-22

**Authors:** Saliha Kevser Kavuncu, Mehmet Yalvac, Alper Basturk

**Affiliations:** 1 Department of Computer Engineering, Graduate School of Natural and Applied Sciences, Erciyes University, 38039 Kayseri, Türkiye; 2 Department of Computer Technologies, Sungurlu Vocational School, Hitit University, 19300 Corum, Türkiye; 3 Department of Anesthesiology and Reanimation, Faculty of Medicine, Hitit University, 19040 Corum, Türkiye; 4 Department of Computer Engineering, Erciyes University, 38039 Kayseri, Türkiye; 5 Artificial Intelligence and Big Data Application and Research Center, Erciyes University, 38039 Kayseri, Türkiye

**Keywords:** anesthesia depth, multitask learning, time–frequency analysis, electroencephalography (EEG), explainable artificial intelligence (XAI), bispectral index (BIS)

## Abstract

**Background:** Electroencephalography (EEG) signals are widely used for monitoring anesthesia depth during surgery. Current commercial indicators are largely closed-source and may reflect dynamic changes with some delay. **Methods:** This study proposes a multitask deep learning model for continuous Bispectral Index (BIS) estimation, binary anesthesia-state classification, and prediction of transitions toward light anesthesia at different time intervals. Dual-channel EEG signals from 5471 surgical cases in the VitalDB dataset were divided into 60 s windows. Short-Time Fourier Transform (STFT) captured instantaneous frequency changes to transform the signal into a two-dimensional map. A ResNet-SE architecture incorporating Squeeze-and-Excitation blocks was used to identify EEG features associated with anesthesia depth. **Results:** A Mean Absolute Error of 3.27 and a Root Mean Square Error of 5.48 were obtained in anesthesia depth estimation. Light anesthesia classification achieved an AUC of 0.99 on the internal test set. **Conclusions:** The proposed multitask model enables the assessment of anesthesia depth and transitions toward light anesthesia using EEG signals.

## 1. Introduction

In anesthesia management, accurately monitoring the patient’s level of consciousness is crucial for patient safety. If a patient wakes up during surgery or is exposed to anesthesia that is too deep, it can lead to serious postoperative complications. Insufficient anesthesia levels can result in a return of consciousness during surgery and psychological trauma [1,2]. Excessive anesthesia, on the other hand, can increase the risk of hypotension, delayed awakening, and postoperative cognitive impairment [3,4].

Various indicators are used in clinical practice to measure levels of consciousness. One of the most commonly used indices, the Bispectral Index (BIS; Aspect Medical Systems, Framingham, MA, USA) [5], converts electroencephalogram (EEG) signals into numerical indices and expresses the level of consciousness as a BIS value between 0 and 100. Generated by a proprietary commercial signal processing algorithm, the BIS is widely accepted as a clinical reference. However, index values are affected by complex brain changes during the deepening of anesthesia and awakening, and calculation delays may occur [6,7]. Such approaches may help clinicians monitor dynamic changes around critical anesthesia levels.

During surgery, the depth of anesthesia should be maintained between 40 and 60 on the BIS scale, which serves as a critical threshold. A BIS value approaching 60 increases the risk of awakening, while a value below 40 poses a risk of excessive sedation. This has led to the development of an extensive body of literature on the analysis of brain activity. In early studies, spectral indices and entropy-based measures were used to understand EEG structure [7,8,9]. Subsequent studies explored nonlinear methods [10]. However, the anesthesia process is dynamic. Therefore, there is a need to develop AI-supported systems capable of accurately monitoring brain activity and predicting upcoming anesthesia-state transitions. These systems may support surgical safety by providing earlier situational awareness regarding dynamic anesthetic shifts.

Raw EEG data may fail to capture important temporal dynamics. This can make it difficult to fully understand temporal details and may lead to limitations in neurophysiological analysis [11,12]. The Short-Time Fourier Transform (STFT), used for time–frequency analysis, provides a rich and detailed temporal and spectral representation of the EEG signal [13,14,15]. Spectral analysis of brain waves may enhance depth of anesthesia monitoring by capturing subtle neurophysiological changes that are not fully reflected by conventional indicators. Our signal-rich approach differs from multimodal models that combine EEG with hemodynamic variables or drug infusion history. Multimodal models typically require specific infusion data or additional sensors to improve accuracy, thus necessitating operating rooms with specialized monitoring infrastructure. In contrast, this study uses an independent EEG-based approach to offer a broader applicability.

The main contributions of the proposed model can be summarized as follows:

1. Anesthesia depth monitoring is a dynamic and holistic process requiring continuous assessment of the patient’s condition and prediction of impending changes. Therefore, a multitask model is proposed that centers on anesthesia depth monitoring and integrates the classification of clinically significant anesthesia states with the prediction of transitions to light anesthesia.
2. In addition to prediction success, SHAP and occlusion-based analyses were performed to interpret model decisions in the time–frequency domain. The analysis suggests that the model’s predictions are associated with patterns observed in the alpha, delta, and theta bands.
3. The developed model was tested with the publicly available NTUH dataset as a pilot external validation dataset. Although its size was limited, the external dataset was collected from a different center using different instruments and clinical protocols. Rather than proving generalizability, it was used as a pilot validation to assess the transferability and robustness of the model and to observe performance consistency. Performance stability was also examined across multiple clinical subgroups.

The rest of the article is organized as follows: Section 2 reviews the current literature and developments in the field of anesthesia depth monitoring. Section 3 details the architecture of the proposed model and the explainability analyses of its predictions. Section 4 reports the performance results of the model. Section 5 interprets the clinical significance of these findings in relation to existing studies. The limitations of the proposed model and future research directions are discussed in Section 6, while Section 7 presents the conclusions and a summary of the main findings.

## 2. Related Work

Initially, research focused on nonlinear parameters that measured signal complexity and irregularity. Methods such as Approximate Entropy (ApEn) and Shannon Entropy have proven successful in determining the depth of anesthesia [16,17]. During this period, the analysis of the depth of anesthesia mainly focused on regression (predicting a continuous index value, usually a BIS score between 0 and 100) and classification (separating the stages of awake, light, general, and deep anesthesia) tasks [3,18]. In subsequent research, machine learning algorithms and advanced feature engineering came to the forefront. By employing the ANFIS–LH architecture on a combined set of 11 spectral, fractal, and entropy-based features, researchers reported a high classification performance of 92.91% [18]. In this process, the use of feature sets designed with expert knowledge instead of raw EEG signals became widespread [19].

The emergence of deep learning has increased the trend toward Convolutional Neural Networks (CNN) for learning directly from raw data with a model. Anesthesia depth was estimated from raw EEG data via regression using the AnesNET architecture developed with the VitalDB dataset. The biggest advantage of this model was that it enabled real-time monitoring by reducing the 30–60 s latency in commercial monitors to 20 ms [6]. To enable learning through the temporal dynamics of EEG signals, subsequent studies began combining CNNs with Long Short-Term Memory (LSTM) units and Attention layers. For instance, a CNN structure similar to Inception modules was combined with a Bidirectional LSTM and an attention layer, achieving an 88.7% accuracy (ACC) in anesthesia level classification [20]. These hybrid models created architectures more robust against noise in the signal and began to yield better results in data with physiological cross-subject variations [14].

In modern architectures, Transformer and Self-Attention structures have begun to be used in anesthesia analysis with EEG signals. Transformer-based models have been proposed to more efficiently process long-term dependencies and spectral characteristics in time series [21,22]. These models used label distribution correction techniques to minimize prediction errors and, in particular, to address the data imbalance problem.

Recent studies have expanded EEG-based anesthesia monitoring toward transition prediction and multitask learning. Deep-ConTrans was proposed for consciousness-transition classification under sedation conditions [23]. DEBP jointly addressed BIS estimation and sedation-state classification within a multitask model [24]. More recently, AnesNet focused on future anesthesia-depth prediction using multimodal intra-operative signals from the VitalDB dataset [25]. These studies demonstrate the growing interest in predictive and transition-aware anesthesia monitoring systems.

Reinforcement Learning (RL) studies aimed at autonomously managing anesthetic drug dosage along with monitoring the depth of anesthesia have also begun to be used in recent years. Models have been developed using hierarchical deep reinforcement learning (DIAPG) to manage propofol and remifentanil infusions with an “autonomous pilot”-like control mechanism. With this approach, both the BIS value was predicted and the doses required to keep the patient at a stable sedation level were successfully optimized [26]. Consistency of findings with physiological data is vital for the safe management of actual anesthesia procedures. Current trends focus on making models clinically interpretable rather than “black boxes”. Attention maps were used to visualize which frequency bands the model focused on, particularly the alpha and delta bands [27].

The primary goal of this study is to monitor anesthesia depth relative to the clinically relevant BIS range of 40–60 and to predict transitions toward light anesthesia. This requires both monitoring the instantaneous BIS value and accurately predicting transitions. The proposed Residual Network–Squeeze-and-Excitation (ResNet–SE)-based multitask model includes a time-to-event prediction structure using 3, 5, and 10 min intervals. It provides the anesthesiologist with information on the current anesthesia status as well as situational awareness of upcoming changes. This may provide additional information for anesthetic management, but it is not intended to replace existing monitors. In addition to the presented performance, neurophysiological signals were also analyzed using SHAP. Such explainability features provide additional support for interpreting the biological plausibility of model predictions during surgery.

## 3. Materials and Methods

### 3.1. Proposed Model and General Workflow

**Figure 1:**
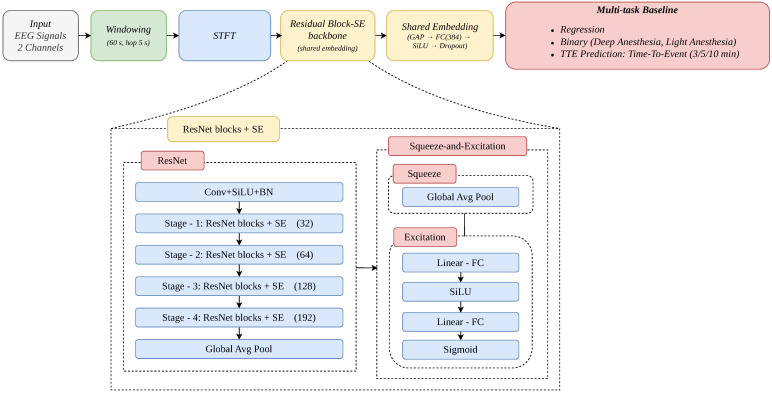
Overview of the proposed multi-tasking ResNet–SE model for anesthesia depth monitoring and transition prediction.

This section discusses the proposed ResNet-SE-based multitask model architecture for monitoring anesthesia depth. The overall structure of the model is shown in Figure 1. In the preprocessing step, raw EEG signals were divided into 60 s windows. These windows were converted into spectrograms in the time–frequency domain using the STFT. The resulting spectrograms were fed as input into a four-stage ResNet architecture, which forms the core of the model. Within this architecture, Squeeze-and-Excitation (SE) blocks were incorporated to recalibrate feature channels potentially associated with anesthesia depth. Batch Normalization layers were used throughout the network to make the model’s training more stable. To better learn complex and nonlinear relationships, the SiLU activation function was applied across the network.

In the next stage of the network, the meaningful features obtained were combined using a Global Average Pooling layer. The resulting features are fed into a 384-unit shared embedding layer. This shared feature pool simultaneously feeds three different output heads that provide the final predictions. This multitask architecture enables the model to estimate the current BIS value, classify anesthesia depth, and analyze potential transitions toward light anesthesia at 3, 5, and 10 min intervals.

### 3.2. EEG Windowing Strategy

In order to apply deep learning models, data often needs to be organized according to a specific data structure and sequence. For this reason, EEG signals recorded or streamed during surgery are not fed into the system as a single continuous stream. Instead, they are divided into fixed-length temporal windows to enable efficient processing of continuous EEG recordings. This method prevents momentary changes in brain waves from being overlooked, thereby ensuring that significant changes are not lost [2,22,28,29].

Segment durations may vary depending on the purpose of the analysis regarding the depth of anesthesia. In clinical practice, BIS devices display the patient’s overall condition by averaging data from the past 60 s [5,10]. Therefore, to align with the device’s operational structure and provide the model with sufficient historical data context, a 60 s window length was chosen for this study. Additionally, the 60 s segment length provided the temporal information required by the model to predict the anesthetic state 3, 5, and 10 min in advance. The segments were divided by shifting them by 5 s each time. Updating the data at short intervals of 5 s allowed for frequent sampling of the signal, ensuring that changes in the anesthesia status were captured more consistently. This also prevented misleading fluctuations that could be caused by transient noise [2].

For training and evaluation, each 60 s EEG segment was treated as an individual model input sample. The *i*-th EEG segment is denoted by $x_{i}$, and the corresponding BIS value is denoted by $y_{i}$:

(1) $$x_{i}\in R^{C\times T},\;y_{i}\in R,$$

where *C* represents the number of EEG channels and *T* denotes the number of temporal samples within the segment.

This window-based representation enabled the model to learn temporal EEG patterns from large-scale intra-operative recordings. Because adjacent segments substantially overlapped, temporal correlation existed between consecutive samples. To prevent patient-level information leakage and overly optimistic performance estimation, all windows belonging to a given surgical case were assigned exclusively to a single subset. Consequently, training, validation, and test splits were performed at the case level rather than at the window level.

### 3.3. Prediction Tasks and Label Definitions

In this study, BIS values obtained from VitalDB were used as surrogate clinical reference labels for model development and evaluation. In addition, proprietary signal processing and temporal smoothing characteristics of BIS should be considered when interpreting model outputs. Here, BIS was considered as an indicator reflecting the patient’s anesthetic status during surgery.

The model was designed as a multitask learning system in which multiple targets were estimated simultaneously from the same EEG window. These tasks included continuous BIS estimation, binary classification of anesthetic states at clinically used BIS thresholds, and time-to-event (TTE) prediction of transitions toward light anesthesia. Let $x_{i}\in R^{C\times T}$ denote the *i*-th EEG window and let $y_{i}\in R$ denote the corresponding BIS value. The indicator function is denoted by I(·), which takes the value 1 if the condition holds and 0 otherwise.

(i) Continuous BIS regression.

For continuous estimation of anesthesia depth, the model produces a continuous BIS estimate:

(2) $${\hat{y}}_{i}^{\mathrm{reg}}\in R.$$

This regression task allows the model to estimate the overall level of anesthesia as a continuous variable rather than restricting the analysis to threshold-based categories only.

(ii) Binary classification tasks (BIN40 and BIN60).

To obtain state predictions at clinically used BIS thresholds, two binary targets were defined. BIN40 corresponds to deep anesthesia (BIS below 40; BIS <40), whereas BIN60 represents light anesthesia (BIS 60 and above; BIS ≥60):

(3) $$y_{i}^{40}=I\left(y_{i}<40\right),\;y_{i}^{60}=I\left(y_{i}\geq 60\right).$$

Here, $y_{i}^{40}$ represents deep anesthesia, whereas $y_{i}^{60}$ represents light anesthesia. These threshold-based tasks enabled the model to distinguish anesthesia levels associated with clinically relevant BIS ranges.

(iii) Time-to-event (TTE) based prediction.

In addition to instantaneous estimation tasks, a TTE approach was used to analyze transitions toward light anesthesia. Accordingly, the first time point at which the BIS value reached or exceeded 60 after remaining below this threshold was defined as a BIS60–up event. Based on this definition, a BIS60–up TTE prediction model was established to investigate EEG patterns observed before transitions toward light anesthesia.

To avoid including EEG activity occurring after the transition, TTE labels were calculated according to the end time of each EEG window rather than the window center. Let $t_{e}$ denote the event time and let $t_{i}^{end}$ denote the end time of the *i*-th EEG window. The remaining time until the event was calculated in minutes as follows:

(4) $${\mathrm{TTE}}_{i}=\frac{t_{e}-t_{i}^{end}}{60}.$$

For predefined intervals h∈{3,5,10} minutes, the corresponding event label was defined as:

(5) $$y_{i,h}^{\mathrm{tte}}=I\left(0<{\mathrm{TTE}}_{i}\leq h\right).$$

Only windows ending before the event time were included in TTE labeling. Windows ending at or after the event time were excluded to avoid the inclusion of post-event EEG activity.

In TTE modeling, cases in which no BIS60–up transition was observed during the recording period were retained in the dataset as negative samples. For cases containing a BIS60–up transition, positive labels were assigned only to windows immediately preceding the event within predefined intervals of 3, 5, or 10 min. Time periods outside these intervals were considered negative if the corresponding windows ended before the event time. This approach allowed both transition and non-transition cases to be included during training and evaluation. To preserve compatibility with clinical practice, the BIS60 threshold and the selected intervals of 3, 5, and 10 min were determined in consultation with a senior anesthesiologist. The BIS40 threshold was used only in classification tasks to define deep anesthesia levels in accordance with the purpose of the model. This multitask structure enables the model to evaluate the current depth of anesthesia while also estimating transitions toward light anesthesia.

### 3.4. Time–Frequency Representation Using STFT

EEG signals exhibit non-stationary dynamic characteristics throughout the onset of anesthesia, its maintenance during surgery, and the stages of awakening. To accurately capture changes in the depth of anesthesia during this process, both temporal and spectral features must be processed simultaneously. To perform this simultaneous analysis, STFT was applied to each EEG segment in the study.

For a discrete time signal x[n], the STFT is defined as:

(6) $$X\left(m,\omega \right)=\sum_{n=-\infty }^{\infty }x\left[n\right]\;w\left[n-m\right]\;e^{-j\omega n},$$

where w[·] denotes the analysis window function, *m* represents the temporal frame index, and ω denotes the angular frequency. STFT coefficients were obtained by performing calculations on each EEG window. Subsequently, maps showing the changes in the time and frequency domains of the signal were created.

**Figure 2:**
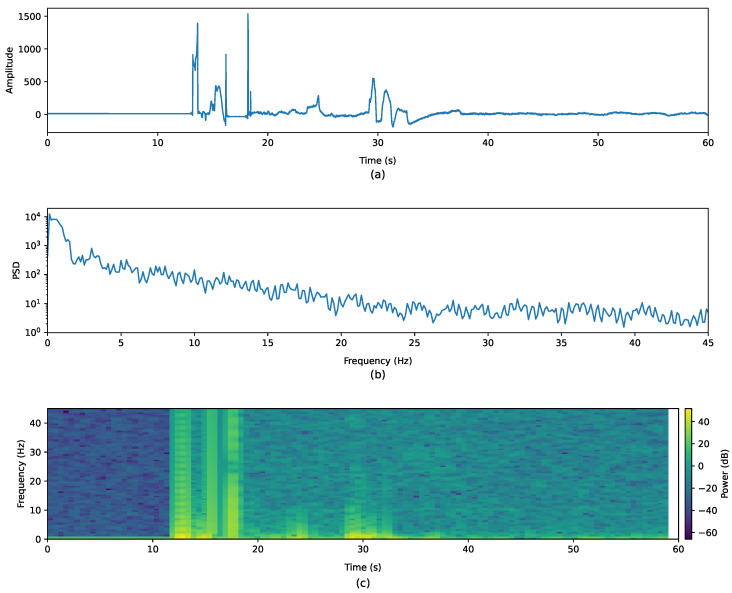
EEG preprocessing and time–frequency conversion process. (**a**) A 60-second segment of the raw frontal EEG signal. (**b**) PSD analysis showing the frequency characteristics of the EEG signal. (**c**) Log–power spectrogram within the 0–45 Hz frequency range used as a two-dimensional input representation for the ResNet architecture.

Figure 2 summarizes the processing steps from raw EEG acquisition to the generation of time–frequency representations used as model inputs. As shown in Figure 2a, complex temporal fluctuations are observed in the raw EEG signal throughout the recording. The Power Spectral Density (PSD) analysis presented in Figure 2b demonstrates that a substantial proportion of signal energy is concentrated in the low-frequency range. To analyze temporal changes in EEG rhythms during anesthesia, EEG segments were transformed into spectrograms using the STFT method. The resulting representation covered the 0–45 Hz frequency range and simultaneously preserved temporal and spectral information, as illustrated in Figure 2c. The resulting STFT time–frequency maps enabled the model to learn temporal and spectral patterns associated with anesthesia depth.

The STFT was computed using a Hann analysis window with a duration of 2 s (256 samples at a sampling rate of 128 Hz) and a hop size of 0.5 s (64 samples), resulting in a 75% overlap between adjacent frames. The FFT length was set to 256. The goal was to reduce high-frequency noise by preserving the 0–45 Hz frequency range. This configuration produced 91 frequency ranges and 117 time frames for each EEG channel.

The STFT was applied to each EEG channel independently, and the resulting time–frequency matrices were concatenated to construct a two-channel input tensor of 2×91×117 dimensions. These data were fed as input into the ResNet–SE model to estimate the depth of anesthesia and to learn anesthesia-related representations.

Before model training and evaluation, EEG windows underwent quality-control procedures. Windows with excessive amplitudes (>1500 μV), abnormally flat signal segments, or a high proportion of missing samples were excluded from further analysis. Following quality control (QC), EEG signals were converted into time–frequency representations using STFT.

The resulting spectrograms were converted into log-power domains using logarithmic scaling. The mean and standard deviation values of the training set were calculated. The data were standardized using these parameters. The same normalization coefficients were also applied to the validation, internal testing, and external validation datasets.

### 3.5. ResNet–SE Backbone and Squeeze-and-Excitation Blocks

The EEG windows, converted into time–frequency representations, were processed using a ResNet architecture enhanced with SE mechanisms. Residual connections enable gradients to propagate effectively across the network, contributing to model training. This allows the model to learn by dynamically weighting and improving existing features rather than directly matching them.

Let $U\in R^{H\times W\times C}$ denote an intermediate feature map, where *H* and *W* represent the spatial dimensions of the spectrogram feature map and *C* denotes the number of channels. The SE mechanism performs channel-wise recalibration in two stages: squeeze and excitation.

Squeeze.

To aggregate spatial information across the feature map, the Global Average Pooling (GAP) operation was applied:

(7) $$z_{c}=\frac{1}{HW}\sum_{i=1}^{H}\sum_{j=1}^{W}U_{c}\left(i,j\right),\;c=1,\ldots ,C.$$

This operation produces a vector $z\in R^{C}$ summarizing global activation statistics across channels.

Excitation.

The obtained channel descriptor was passed through fully connected layers and nonlinear activation functions to generate channel-wise scaling coefficients:

(8) $$s=\sigma \left(W_{2}\;\delta \left(W_{1}z\right)\right),$$

where $W_{1}$ and $W_{2}$ denote learnable weight matrices, δ(·) represents the SiLU activation function, and σ(·) denotes the sigmoid function. The resulting vector $s\in {\left(0,1\right)}^{C}$ contains channel-wise scaling coefficients.

Feature recalibration.

Finally, the original feature map was recalibrated through channel-wise multiplication:

(9) $${\tilde{U}}_{c}=s_{c}\cdot U_{c},\;c=1,\ldots ,C,$$

where $\tilde{U}$ denotes the SE-enhanced feature map.

In the proposed architecture, SE blocks are incorporated at each stage of the ResNet backbone. This allows for reweighting of channel responses and a much more efficient representation of frequency components. To improve the stability of the training process, Batch Normalization is applied across the network, and SiLU is chosen as the activation function.

In the final stage, the extracted high-level features are combined using Global Averaging Pooling to create a 384-dimensional common representation space. This shared representation was subsequently used for BIS regression, anesthetic state classification, and TTE prediction tasks at different intervals.

### 3.6. Computational Cost and Inference Latency

To evaluate the clinical feasibility of the proposed ResNet–SE model under operating room conditions, memory requirements and inference latency were analyzed across different hardware configurations. The architecture contains 6,799,753 trainable parameters, corresponding to approximately 25.94 MB in FP32 precision. This relatively low memory requirement suggests that the proposed approach can be deployed without requiring high-cost computing infrastructure.

Inference latency was evaluated using a batch size of 1 to simulate a real-time clinical data stream. Warm-up iterations were performed before timed runs in order to exclude initialization overhead from the measurements. Performance was reported using mean ± standard deviation and p95 latency values.

On CPU with 8 threads, the model processed a single 60 s STFT window in 3.320 ± 0.593 ms, with a p95 latency of 4.400 ms. To evaluate performance under more limited computational conditions, a single-thread CPU configuration was also tested. In this setting, latency increased to 7.137 ± 0.078 ms, with a p95 latency of 7.267 ms. On an NVIDIA RTX 4090 Laptop GPU (NVIDIA Corporation, Santa Clara, CA, USA), inference time decreased to 1.131 ± 0.030 ms, with a p95 latency of 1.186 ms. Detailed latency measurements for the evaluated hardware configurations are presented in Table 1.

These measurements only show the model inference time. In a real operating room setting, the total system latency depends on: EEG signal acquisition, transient data buffering, and STFT conversion processes. However, even the highest latency value measured in the system remained well below the 5-s update interval determined in this study. These findings suggest computational feasibility for real-time anesthesia monitoring, as the computational load introduced by the proposed model remains low relative to the clinical data update cycle.

### 3.7. Subgroup Analysis

Subgroup analysis was conducted to assess the consistency of the model across different patient characteristics and clinical situations. Performance on the test set was evaluated across age (<60 and ≥60 years), sex (female and male), American Society of Anesthesiologists (ASA) physical status (ASA 1–2 and ASA 3–4), different surgical categories, and predefined demographic characteristics.

Subgroup performance for both deep anesthesia and light anesthesia classification tasks was examined through Area Under the Curve (AUC) value. The trained model was applied to all subgroups without parameter changes or fine-tuning. The aim of this analysis was to examine whether model performance remained stable across different patient populations and clinical settings.

### 3.8. Explainability Analysis

To investigate the model predictions and identify the time–frequency components influencing the outputs, SHAP (SHapley Additive exPlanations) analysis was performed [30]. SHAP is a game-theory-based feature attribution method that estimates the contribution of individual input features to the final model output.

Let f(x) denote the output of the trained model for a given input *x*, corresponding to either the predicted BIS value or the probability score of a classification or TTE outcome. SHAP approximates f(x) using an additive attribution model defined as

(10) $$f\left(x\right)\approx \phi_{0}+\sum_{k=1}^{M}\phi_{k},$$

where $\phi_{0}$ represents the expected model output over a background distribution and $\phi_{k}$ denotes the SHAP value associated with the *k*-th feature among *M* input features. A positive $\phi_{k}$ indicates that the corresponding feature increases the model output relative to the baseline, whereas a negative value indicates a decreasing contribution.

In this study, SHAP analysis was applied to STFT-based time–frequency representations of EEG signals to identify spectrotemporal regions contributing most strongly to anesthesia depth estimation and transitions toward light anesthesia. The obtained SHAP values were normalized across time and frequency dimensions, aggregated, and interpreted according to established EEG frequency bands.

To further examine the robustness of the SHAP findings, an additional frequency band masking (occlusion) analysis was performed. In this approach, selected EEG frequency bands were masked by setting the corresponding STFT coefficients to zero while preserving the remaining components of the input signal. Let $x^{\left(-b\right)}$ denote the input after masking frequency band *b*. The effect of masking was quantified by measuring the change in model output:

(11) $$\Delta f_{b}=f\left(x\right)-f\left(x^{\left(-b\right)}\right),$$

where larger values of $\Delta f_{b}$ indicate greater sensitivity of the model to the masked frequency band.

Decreases in prediction performance observed after masking specific frequency bands were interpreted together with SHAP results to evaluate the physiological relevance of the highlighted spectrotemporal EEG patterns.

### 3.9. Pilot External Validation

For the pilot external evaluation, a publicly available dataset containing raw EEG recordings and BIS values from 24 surgical cases from National Taiwan University Hospital (NTUH) [31] was used. Publicly available anesthesia datasets containing both EEG recordings and BIS values are limited. Although the number of cases in the NTUH dataset was limited, it provided us with a useful pilot external validation dataset to test the model’s ability to generalize across various clinical populations.

The EEG recordings from the external dataset were subjected to the same preprocessing applied to the internal dataset. The EEG signals were divided into 60 s windows and then converted to time–frequency features. The model trained on the internal dataset was applied directly to the external dataset without any fine-tuning or parameter changes.

In the pilot external evaluation, continuous BIS regression, binary anesthesia-state classification, and transition prediction toward light anesthesia were examined. This external evaluation is not intended to replace large-scale multicenter studies. It is considered merely a preliminary test demonstrating the robustness and transferability of the model in different clinical settings.

## 4. Results

This section presents the performance of the proposed multitask ResNet-SE model in continuous BIS regression, threshold-based anesthesia state classification, and transition prediction across different time intervals. Furthermore, subgroup analyses, explainability findings, and pilot external validation results are reported to demonstrate the model’s consistency under various clinical conditions.

### 4.1. Dataset and Data Selection

The data used in this study were obtained from the open-access VitalDB [32] database containing high-resolution, multiparameter physiological recordings of surgical patients at Seoul National University Hospital. This comprehensive dataset, collected simultaneously from multiple medical devices using Vital Recorder software (Seoul National University College of Medicine, Seoul, Republic of Korea), covers 6388 cases. It also includes intra-operative recordings of surgical cases, including dual-channel EEG signals. 5566 of the analyzed cases have both high-resolution EEG data and BIS measurements.

QC procedures were applied during preprocessing. Cases with signal loss, excessive noise or insufficient recording time were excluded from the analysis. After QC, a total of 5471 surgical cases were used for model development.

A total of 3,370,330 EEG windows were generated for analysis. To prevent information leakage, dataset segmentation was performed at the case level. All windows belonging to the same surgical case were assigned to a single subset, ensuring complete separation between training, validation, and test data. The training set consisted of 4103 cases and 2,536,299 windows, the validation set consisted of 821 cases and 520,203 windows, and the test set consisted of 547 cases and 313,828 windows.

For external evaluation, an independent dataset provided by National Taiwan University Hospital (NTUH) was used as the pilot external validation dataset. The NTUH dataset consisted of 24 surgical cases and 1901 EEG windows.

### 4.2. Continuous BIS Prediction and Classification Results

The model obtained a Mean Absolute Error (MAE) value of 3.27. This value indicates an average difference of approximately 3 points between the predicted BIS value and the reference BIS value. The Root Mean Square Error (RMSE) was 5.48. Since the RMSE value is calculated by squaring the error, it is more sensitive to large deviations. The difference between MAE and RMSE shows that the model generally produces close BIS predictions, but larger errors may occur in cases with abrupt signal changes or noisy segments. The coefficient of determination ($R^{2}$) was calculated as 0.84. This result shows that the model captures approximately 84% of the variance in BIS values.

**Figure 3:**
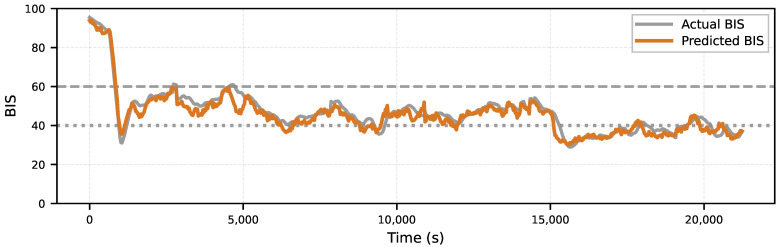
Temporal tracking of BIS predictions and reference BIS values in a sample case from the VitalDB dataset. The gray line represents the reference BIS values, whereas the orange line represents the model-predicted BIS values. The model output closely follows the reference BIS signal throughout the recording period. This figure represents a single illustrative test case.

The performance of the ResNet–SE model in monitoring BIS values is shown in Figure 3 using a sample EEG signal selected from the dataset. The model consistently adapted to BIS changes, showing good agreement with the reference BIS signal.

To complement the illustrative example shown in Figure 3, case-level regression performance was also tested across the internal test set. Across 547 test cases, the median MAE was found to be 3.58 (Interquartile Range (IQR): 2.70–4.68) and the median RMSE was 5.12 (IQR: 3.92–7.04).

**Figure 4:**
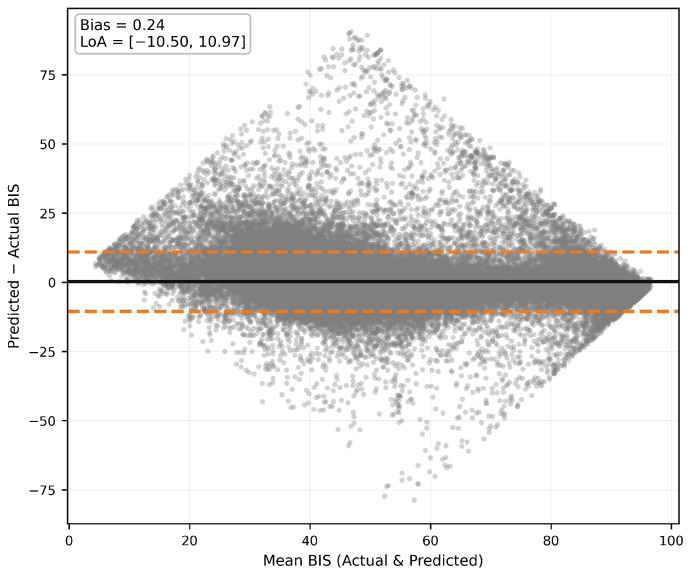
Bland–Altman plot for the internal test set showing the distribution of differences between the estimated BIS and reference BIS values. The solid black line represents the mean bias, whereas the dashed orange lines indicate the upper and lower LoA. All samples from the internal test set were included in this analysis.

The agreement between the predicted BIS values and the reference measurements was further evaluated using a Bland–Altman analysis (Figure 4). The mean deviation of 0.24 indicates that there is no significant systematic bias in the model. Furthermore, the 95% Limits of Agreement (LoA) ranging from −10.50 to 10.97 show that the prediction differences mostly remain within a narrow range. The clustering of data points, particularly around the zero deviation line in the clinically significant anesthesia range (BIS 40–60), supports the consistency of the model predictions.

The internal evaluation results in Table 2 and Table 3 show that the multitask ResNet–SE model performed consistently across different anesthesia-related classification tasks. The similarity of validation and internal test results in the deep anesthesia classification may indicate low variability between data sets. For light anesthesia, high AUC and specificity values suggest effective separation of anesthesia states with few false-positive predictions. Overall, the internal results indicate that STFT-based time–frequency representations combined with SE-enhanced convolutional layers provide a stable structure for modeling EEG patterns across different anesthesia levels.

**Figure 5:**
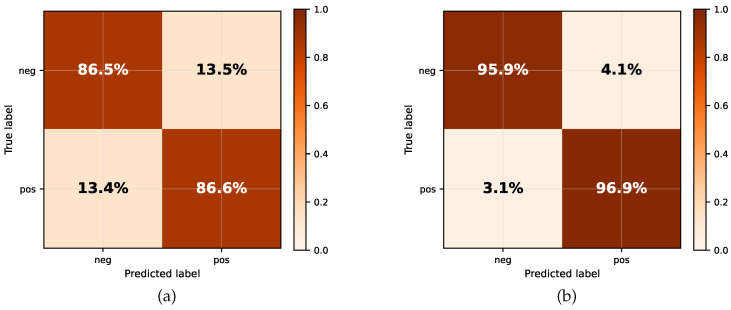
Percentage confusion matrices for internal test classification tasks: (**a**) deep anesthesia classification and (**b**) light anesthesia classification.

**Figure 6:**
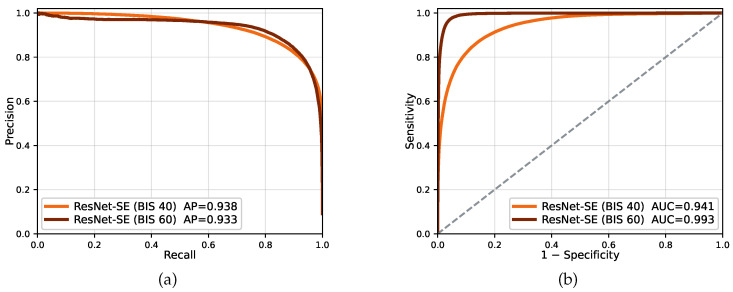
Precision–Recall and ROC curves for BIS threshold classification tasks on the internal test set. (**a**) Precision–Recall curves for deep anesthesia and light anesthesia. (**b**) ROC curves for deep anesthesia and light anesthesia. The dashed diagonal line indicates random classifier performance.

The classification results from the internal test set are presented in Figure 5 and Figure 6. The confusion matrices in Figure 5 show that the proposed model distinguishes between deep and light anesthesia states with consistent performance. In both binary classification tasks, most samples were assigned to the correct class (Figure 5a,b). Specifically, the true positive rate for light anesthesia reached 96.9% (Figure 5b), indicating high sensitivity for transitions toward lighter anesthesia.

The Precision–Recall curves in Figure 6a show a stable balance between precision and recall, while the ROC curves in Figure 6b remain close to the upper-left region. Taken together, these performance trajectories demonstrate the model’s reliability in threshold-based classification, offering consistent metrics for intra-operative tracking.

### 4.3. Prediction of Transitions Toward Light Anesthesia

This section evaluates the model’s performance in predicting transitions toward light anesthesia, defined as the point at which the BIS value first reaches or exceeds the threshold of 60 (BIS60–up). An independent internal test dataset consisting of 547 surgical cases was used for evaluation. Prediction performance for transitions toward light anesthesia is summarized in Table 4. The AUC values obtained for the first BIS60–up transition were 0.94, 0.91, and 0.85 for the 3, 5, and 10 min prediction intervals, respectively. A gradual decrease in performance was observed as the prediction interval increased. This finding suggests that EEG patterns become less directly associated with the transition point at longer temporal distances.

Because the model analyzes highly overlapping EEG windows, consecutive high-risk predictions may occur during the same transition period. To reduce repeated alerts originating from the same event, temporally adjacent predictions were grouped into single alert episodes. In this way, repeated detections associated with the same transition process were evaluated together within a unified episode structure. The performance of this strategy is summarized in Table 5. The model generated at least one alert episode before the transition toward light anesthesia in 534 cases for the 10 min prediction interval. Across different prediction intervals, the median alert episode frequency ranged between 3.92 and 4.52 episodes per hour. Wider prediction intervals increased the number of detected transitions, but also resulted in a proportional increase in alert frequency.

**Figure 7:**
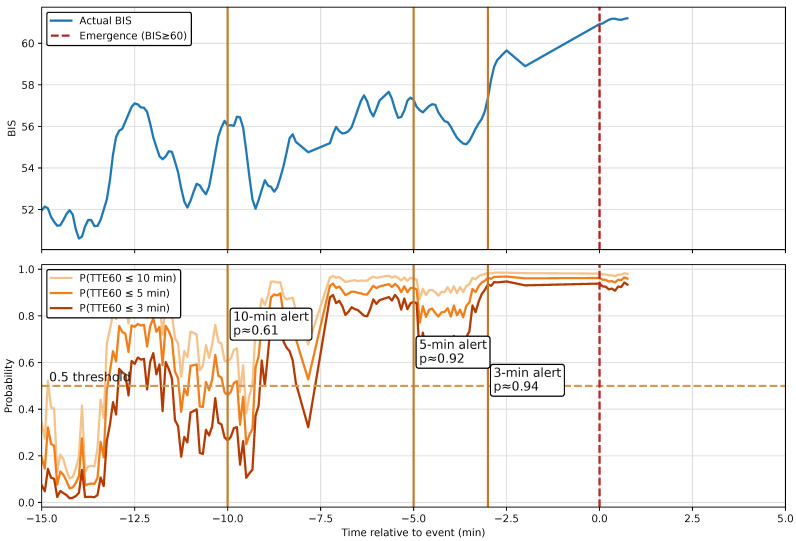
Case 1205–BIS trajectory and alert episodes preceding the transition toward light anesthesia. The red dashed vertical line indicates the BIS60–up event time point.

To further examine the temporal behavior of the alerts, Case 1205 is presented in Figure 7. As the transition toward light anesthesia approaches, alerts appear sequentially across different prediction intervals. The first alert is observed approximately 10 min before the threshold crossing, followed by additional alerts within the 5 and 3 min intervals. This pattern illustrates the model’s ability to capture EEG changes occurring at different temporal distances before the transition.

### 4.4. Impact of Squeeze-and-Excitation Blocks on Model Performance

To evaluate the contribution of SE blocks, the proposed ResNet–SE architecture was compared with a baseline ResNet model in which the SE blocks were removed. Both models were trained and evaluated under identical experimental conditions. As shown in Table 6, the inclusion of SE blocks improved performance across all prediction tasks.

For continuous BIS estimation, the MAE decreased from 4.08 to 3.27, while the RMSE decreased from 6.13 to 5.48. Improvements were also observed in both deep and light anesthesia classification tasks, where higher AUC values were achieved with the ResNet–SE model. A similar trend was observed for transition prediction, with performance gains across all tasks.

### 4.5. Subgroup Performance Analysis

**Figure 8:**
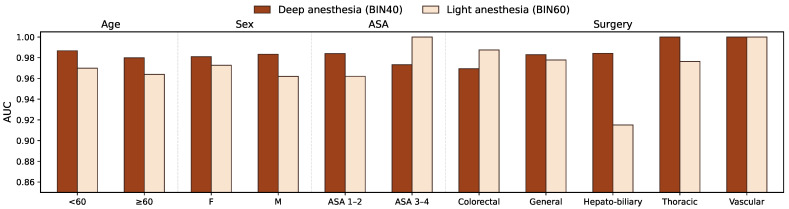
Subgroup analyses showing model performance under diverse patient profiles and clinical conditions. Relevant AUC values are presented across age, sex, ASA physical status, and surgical categories.

The classification performance across demographic and clinical subgroups is summarized in Figure 8. Overall, AUC values remained relatively stable across different subgroups, including age, sex, ASA physical status, and surgical categories, for both deep and light anesthesia classification tasks. No marked performance decrease was observed in any subgroup. Minor differences were noted between surgical categories and ASA groups, which may reflect variability in patient characteristics and anesthesia management practices. Achieving similar success rates across subgroups may demonstrate the model’s consistency across different patient structures and clinical situations.

### 4.6. Neurophysiological Explainability

SHAP analysis was performed to examine how the model’s prediction of anesthesia status could be explained. SHAP values provide an attribution-based explanation of model behavior; however, they do not establish causal relationships between specific EEG patterns and model outputs, particularly in highly correlated representations such as STFT maps. Overall, the results showed a consistent hierarchy among EEG frequency bands across different transition states. Delta and alpha bands were associated with larger SHAP contributions, while theta and beta bands also showed notable contributions. These observations were broadly consistent with EEG patterns previously reported in studies of anesthesia depth.

**Figure 9:**
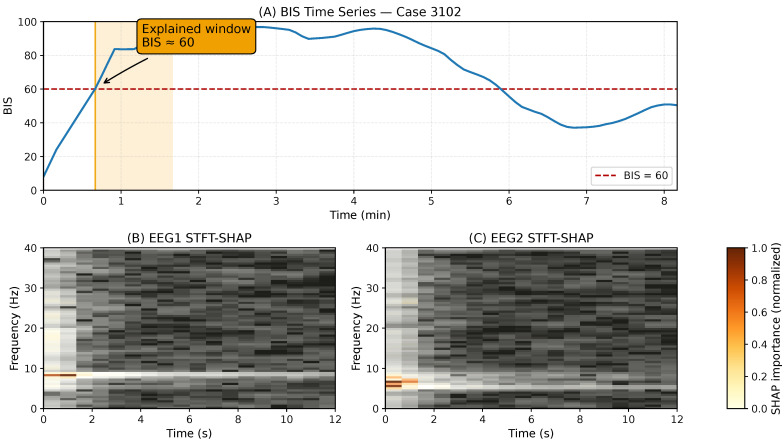
Local SHAP explanations for Case 3102 during the transition toward light anesthesia. (**A**) BIS time series for Case 3102, where the red dashed horizontal line indicates the BIS 60 threshold and the shaded region indicates the explained time window. (**B**) EEG1 STFT–SHAP map. (**C**) EEG2 STFT–SHAP map. The color intensity in the SHAP maps represents normalized SHAP contribution magnitude.

At the case level, local SHAP explanations for the BIS60–up transition in Case 3102 suggested greater importance of low-frequency and alpha-band dynamics. As illustrated in Figure 9, regions with high SHAP contributions were observed before the actual clinical threshold crossing occurred. These attribution patterns indicate that the model assigned greater importance to specific EEG time–frequency regions during this period; however, they should not be interpreted as evidence that these EEG components causally triggered the prediction or the subsequent transition toward light anesthesia. The identified time–frequency patterns were additionally reviewed by an anesthesiology expert and were considered broadly compatible with neurophysiological patterns commonly described in the anesthesia literature.

**Figure 10:**
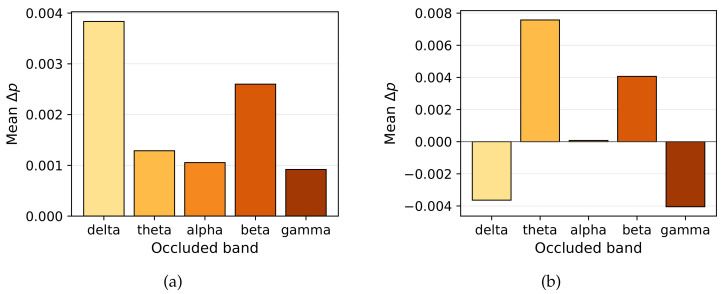
Occlusion sensitivity analysis across EEG frequency bands. (**a**) Deep anesthesia: masking the delta band produced the largest prediction change. (**b**) Light anesthesia: masking the theta band produced the largest prediction change.

To further validate the model decision process, occlusion sensitivity analysis was performed by selectively masking EEG frequency bands and measuring the resulting change in prediction probability. For deep anesthesia detection, the largest prediction change was observed after masking the delta band, suggesting that model predictions were relatively sensitive to information contained within this frequency range (Figure 10a). In contrast, masking the theta band produced the largest prediction change during light anesthesia prediction, as shown in Figure 10b.

Additionally, masking delta activity during awake or light anesthesia periods increased the predicted probability of light anesthesia. This finding suggests that delta-band information influenced model predictions in these examples. Overall, the occlusion results were broadly consistent with the attribution patterns observed in the SHAP analysis. Nevertheless, both SHAP and occlusion analyses provide associative explanations of model behavior and do not establish causal relationships between specific EEG frequency bands and model decisions.

### 4.7. Comparison with Previous Studies

Table 7 summarizes the performance of the proposed ResNet–SE model in comparison with previously reported studies for anesthesia depth estimation and classification. Comparisons were performed using commonly reported regression metrics (RMSE and MAE) and classification metrics (ACC and AUC). Entries marked with “–” indicate that the corresponding metric was not reported in the original study.

When regression performance was examined, the proposed model achieved an MAE value of 3.27, representing a relatively low estimation error compared with previously reported studies. In studies performed on datasets similar to ours, MAE values around 4.70 have been reported [2]. The obtained RMSE value also remained within the range reported in related studies [27]. For classification tasks, the proposed model achieved an ACC value of 0.96 and an AUC value of 0.99 for light anesthesia detection, indicating stable classification performance across different anesthesia states.

Several previous studies were conducted using relatively limited or single-center datasets [20,33]. In contrast, the present study utilized 5471 surgical cases from the VitalDB database and additionally included a pilot external evaluation using the NTUH dataset.

Recent studies have explored multitask BIS estimation, sedation-state classification, and consciousness-transition prediction [23,24,25]. Deep-ConTrans reported AUC values of 0.97 and 0.95 for consciousness-transition classification [23], whereas DEBP achieved MAE values of 4.75 and 4.39 together with AUC values of 0.96 and 0.93 for BIS estimation and sedation-state classification [24]. AnesNet reported an MAE of 4.90 and an AUC of 0.89 for future anesthesia-depth prediction using multimodal perioperative signals [25]. The studies summarized in Table 7 demonstrate the growing interest in multitask and predictive approaches for anesthesia monitoring. The proposed model achieved an MAE of 3.27 and an AUC of 0.99 while integrating BIS estimation, anesthesia-state classification, and transition prediction toward light anesthesia within a unified EEG-based multitask model.

Although the external validation dataset was limited in size, this evaluation provided preliminary evidence that the proposed model preserved relatively stable predictive behavior under different acquisition conditions and clinical protocols.

**Table 7:** Comparison with previous studies for BIS estimation and anesthesia depth classification.

Source	Method	Dataset	RMSE	MAE	ACC	AUC
[20]	CNN–BiLSTM	Private dataset (n=176)	5.59	4.30	0.88	0.81
[34]	AdaRNN–KD	VitalDB & In-house (n=332&44)	9.40 & 10.44	7.45 & 8.76	–	–
[33]	DRSN–CW	Private dataset (n=18)	–	–	0.95	–
[27]	LSTM + Attn.	VitalDB & AMC (n=5566 & 90)	5.07 & 8.61	–	0.95 & 0.89	0.99 & 0.93
[2]	FEHANet	VitalDB (n=176)	6.18	4.70	0.89	0.83
[23]	Deep-ConTrans	Propofol & Midazolam EEG (n=9&10)	–	–	0.93 & 0.95	0.97 & 0.95
[24]	DEBP	Propofol & Desflurane EEG (n=76&70)	6.72 & 6.08	4.75 & 4.39	0.94 & 0.94	0.96 & 0.93
[25]	AnesNet	VitalDB (n=2803)	–	4.90	–	0.89
**Proposed**	**ResNet–SE**	**VitalDB & NTUH (** ***n*** **= 5471 & 24)**	**5.48 & 6.63**	**3.27 & 4.69**	**0.96 & –**	**0.99 & 0.95**

### 4.8. Pilot External Evaluation for Transferability

The pilot external evaluation results summarized in Table 8 show the model’s performance with data obtained from different medical centers and data collection settings. In the regression task, the model obtained a MAE of 4.69 and a RMSE of 6.63. These error rates indicate that the model exhibits overall agreement with the internal evaluation despite differences in recording conditions.

In the classification tasks for deep and light anesthesia detection, AUC values of 0.91 and 0.95 were obtained, respectively. These values suggest that the learned EEG representations are relatively stable across datasets obtained under different clinical protocols and patient characteristics. Therefore, these results should be interpreted as preliminary evidence of transferability rather than definitive proof of generalizability.

For transition prediction, the TTE formulation targeting the first BIS60–up event was also evaluated. AUC values of 0.82, 0.81, and 0.78 were obtained for the 3, 5, and 10 min prediction intervals, respectively. The gradual decrease in performance with increasing temporal distance is consistent with the higher uncertainty associated with predictions made further from the transition point.

Given the limited size of the external dataset, this evaluation was not intended to replace large multicenter validation studies. Instead, it provides preliminary evidence that the proposed approach preserves similar predictive behavior under different clinical and technical conditions.

## 5. Discussion

This study presents an approach to monitoring the depth of anesthesia by combining the time–frequency dynamics of EEG signals with a multitask deep learning architecture (Figure 1). The multitask approach enables the learning of shared representations among relevant clinical objectives. The ResNet-SE structure uses task-specific output heads and learns shared representations across tasks. This structure prevents the model from overfitting to a single task while also increasing temporal consistency. The focus has been on dynamic neurophysiological explanation supported by subsequent explainability analyses such as SHAP, not just validation metrics [30].

### 5.1. Methodological Rationale and Performance Success

The obtained MAE value of 3.27 and RMSE of 5.48 indicate that the proposed model achieved stable and competitive performance in intra-operative anesthesia depth estimation (Table 7). The results were comparable to, and in some metrics higher than, those reported in representative studies on BIS estimation and anesthesia-state classification summarized in Table 7. As summarized in Table 7, the proposed model demonstrated performance comparable to or higher than several recently reported approaches, including Deep-ConTrans [23], DEBP [24], and AnesNet [25]. The proposed model achieved lower MAE values than DEBP and AnesNet while maintaining strong classification performance across both internal and external evaluations. The observed performance of the proposed model compared with raw EEG-based or static spectral approaches may be related to the time–frequency representation provided by STFT [35]. EEG signals under anesthesia are dynamic, and therefore, neurophysiological changes need to be analyzed. In our model, we transformed the 1-dimensional signal into a 2-dimensional spectrogram via STFT, turning the process into a visual pattern recognition problem suited to our architecture. Another key factor is the model’s ability to dynamically focus on channel based features through SE blocks. As shown in Table 6, the inclusion of SE blocks was associated with improved performance in BIS estimation, anesthesia state classification, and the prediction of transitions toward light anesthesia. More pronounced improvements were observed for the transition prediction tasks. These results suggest that SE blocks may be a beneficial component of the proposed architecture.

Compared to hybrid BiLSTM-based models or Transformer based architectures [20,21], the low error rate of our model may provide additional support for dose adjustment decisions in anesthesia management. The Bland–Altman analysis also supports this stability (Figure 4). The mean deviation is low. The error does not accumulate within a specific BIS range. This suggests that the model does not produce consistently high or low predictions, which could be risky in practice.

### 5.2. Transition Prediction and Clinical Interpretation

In this study, the commercial BIS index was used as a reference label for training the model. Given the patented (closed-box) nature of the BIS algorithm and the potential for errors due to various factors, the risk of the deep learning model learning these errors can be considered a limitation. However, the proposed model helps mitigate this limitation through two main aspects. First, the model provides information about transitions toward light anesthesia before the BIS threshold is reached. The TTE module estimates the probability of transition toward light anesthesia within 3, 5, and 10 min intervals. This may provide additional information before BIS threshold transitions occur, although prospective clinical validation is required to determine its effect on clinical decisions. Secondly, while the BIS algorithm does not explain the reason for the decision, our model shows which frequency band (e.g., Delta or Alpha activity) the decision is based on using SHAP analysis. This provides clinicians with information about which EEG regions influenced the model output. This may provide additional interpretability by showing whether the model relies on physiologically meaningful EEG patterns rather than nonspecific signal changes.

In clinical practice, maintaining a BIS value between 40 and 60 prevents risks that may occur during surgery and affects surgical safety. Our model performed well at these clinically critical thresholds. It achieved an AUC of 0.99 for the BIN60 task (light anesthesia). It also achieved an AUC of 0.94 for the BIN40 task (deep anesthesia) (Table 2 and Table 3). In addition, the model predicted the risk of BIS60–up consistently in both datasets (Table 4 and Table 8).

### 5.3. Alarm Strategy and Practical Workflow Integration

To ensure applicability to real clinical settings, an episode based alarm strategy was included to the model. It was designed to reduce repeated alerts from overlapping windows while preserving frequent temporal monitoring. The model addresses potential alarm intensity with a specific grouping mechanism (Table 5). This design ensures that the clinician is appropriately alerted to neurophysiological changes without being affected by transient fluctuations. The 10-min interval is intended to give clinicians enough time for early adjustments during surgery. Earlier identification of transitions toward light anesthesia may provide additional temporal context for anesthetic management. However, prospective clinical studies are required to determine whether such predictions improve clinical outcomes. This model extends conventional retrospective BIS estimation by incorporating transition-oriented temporal risk analysis that allows for earlier and more controlled anesthesia adjustments. Preliminary qualitative interpretation by the collaborating anesthesiologist suggested that the generated alerts were temporally consistent with clinically relevant EEG changes.

### 5.4. Robustness, Transferability, and Hardware Independence

Comparing the findings from the internal VitalDB test set with the external NTUH pilot validation set reveals a consistent trend (Table 8). Performance in the NTUH dataset (MAE: 4.69) showed a slight decrease compared to the internal test set (MAE: 3.27), as expected due to field shift resulting from different recording hardware and clinical protocols. Furthermore, the results from the pilot dataset demonstrated competitive performance with literature results. However, the model maintained a high classification ability (AUC > 0.90) even with this limited dataset of 24 patients (Table 3 and Table 8). These results should be interpreted as preliminary evidence regarding transferability. The preservation of performance without additional fine-tuning suggests that the learned time–frequency representations remained relatively stable across datasets. Overall, these findings should be interpreted as preliminary evidence regarding the model’s transferability and its potential as a decision support model.

Although the exclusion of drug concentrations and surgical stimulation data might initially appear to be a methodological limitation, this design choice was deliberately made to facilitate clinical universality. Our approach aims to enable easier adaptation to diverse clinical environments without relying on specialized equipment like Target Controlled Infusion (TCI) pumps, which necessitate complex data synchronization and exhibit limitations due to inter-patient variability [36]. By relying exclusively on two-channel EEG, we minimize technical requirements and reduce dependency on multimodal monitoring systems [37]. This strategy is supported by recent literature emphasizing that multimodal physiological signals are secondary and highly variable, whereas EEG provides a direct neurophysiological signal for depth of anesthesia (DoA) estimation [19,22]. Our methodology is grounded in the principle that the ultimate determinant of the anesthetic state is not the administered dose itself, but the direct neurophysiological response of the brain. The results demonstrate that the proposed model is capable of reflecting these neurophysiological dynamics directly from EEG signals, independently of external drug administration metrics.

### 5.5. Neurophysiological Explainability and Feature Importance

Model performance is influenced by physiological changes; therefore, understanding which EEG patterns are associated with model predictions is important. However, studies focusing on explainability in anesthesia monitoring remain limited. Our SHAP analyses suggested that delta- and alpha-band regions were associated with relatively larger attribution values than other frequency bands. These observations were broadly consistent with EEG patterns previously reported in studies of anesthesia depth [38] (Figure 9). In addition, theta-band regions showed relatively higher attribution values during transitions toward lighter anesthesia, which was generally compatible with previous findings [37]. Overall, the SHAP and occlusion results were broadly consistent with anesthesia-related EEG patterns reported in the literature [37] (Figure 10).

## 6. Limitations and Future Studies

One of the main limitations of this study is the relatively small size of the external validation dataset (NTUH, 24 surgical cases). This dataset is substantially smaller than the VitalDB dataset (5471 cases) used for model training and internal evaluation. The limited availability of publicly accessible datasets containing both raw EEG recordings and corresponding BIS labels in the field of anesthesia motivated the use of this dataset. Nevertheless, the model also maintained its classification performance in the external dataset, achieving an AUC value of 0.95 for light anesthesia detection. This result suggests that the model may be usable under different recording conditions. However, since the external evaluation was performed on a limited number of cases, these results should be interpreted as preliminary findings. Further validation studies with larger datasets are needed to more reliably evaluate the model’s performance across different centers and patient populations.

Another limitation of our study is that the current model relies solely on EEG recordings and does not include additional physiological or pharmacological data. Although using only EEG facilitates hardware-independent operation and setup, anesthesia management in clinical practice is inherently multimodal. Therefore, subsequent studies could examine the inclusion of additional inputs, such as drug history or hemodynamic variables, into the system, especially during induction and recovery phases where physiological balance can change rapidly [21,34]. Such additions can provide complementary information without disrupting the method, which is primarily based on EEG.

Finally, although the measured model inference latency was low (Table 1), total deployment latency in real operating room environments may also depend on EEG acquisition, temporary buffering, and STFT preprocessing performed on embedded monitoring hardware. Future work will therefore investigate lightweight preprocessing strategies and model compression methods, including pruning and quantization, to facilitate deployment on portable or resource-limited monitoring systems.

## 7. Conclusions

In this study, a multitasking and explainable deep learning model using time–frequency analysis of EEG signals was developed. The proposed ResNet–SE model performed continuous prediction of BIS values, as well as identification of clinically significant anesthesia states and evaluation of impending transitions to light anesthesia within the same model. Results obtained on the VitalDB dataset showed that the model exhibited successful and consistent performance in both regression and classification tasks. A MAE value of 3.27 was obtained in BIS prediction, while the AUC value reached 0.99 in light anesthesia classification. Furthermore, it evaluated BIS values at different prediction times, 3, 5, and 10 min, without exceeding the 60 threshold, providing additional information regarding impending light anesthesia periods.

SHAP and occlusion analyses revealed that decisions may be largely related to neurophysiologically significant EEG changes observed in the delta, alpha, and theta bands. Subgroup analyses performed across different age groups, ASA classes, and surgical categories showed that overall performance was maintained. Furthermore, a pilot external evaluation conducted on the NTUH dataset revealed that the model could exhibit similar trends under different recording conditions.

In conclusion, EEG-based deep learning methods offer an interpretable and computationally low-cost approach to assessing anesthesia depth. However, larger, multicenter prospective studies are needed to more comprehensively demonstrate the reliability and generalizability of the method in different clinical settings.

## Figures and Tables

**Table 1:** Inference latency per 60 s STFT window with batch size 1.

Platform	Configuration	Mean ± SD (ms)	p95 Latency (ms)
CPU	8 threads	3.320 ± 0.593	4.400
CPU (low-power setting)	1 thread	7.137 ± 0.078	7.267
GPU	NVIDIA RTX 4090 Laptop	1.131 ± 0.030	1.186

**Table 2:** Internal evaluation performance for deep anesthesia classification.

Data Split	AUC	ACC	Sensitivity	Specificity	F1-Score
Training Set	0.92	0.87	0.86	0.88	0.87
Validation Set	0.94	0.86	0.86	0.86	0.86
Internal Test Set	0.94	0.86	0.86	0.86	0.86

**Table 3:** Internal evaluation performance for light anesthesia classification.

Data Split	AUC	ACC	Sensitivity	Specificity	F1-Score
Training Set	0.99	0.93	0.93	0.93	0.93
Validation Set	0.99	0.95	0.95	0.95	0.95
Internal Test Set	0.99	0.96	0.97	0.96	0.96

**Table 4:** Prediction performance for transitions toward light anesthesia.

Task	TTE3 AUC	TTE5 AUC	TTE10 AUC
Light anesthesia transition prediction	0.94	0.91	0.85

**Table 5:** Cases evaluated for alert episode analysis (n = 547 test cases).

Transition	Horizon (min)	Cases with ≥1 Alert Episode	Episodes/Hour Median [IQR]
Light anesthesia transition prediction	3	515/547	3.92 [2.21–5.07]
Light anesthesia transition prediction	5	521/547	4.06 [2.49–5.26]
Light anesthesia transition prediction	10	534/547	4.52 [3.33–5.47]

**Table 6:** Impact of Squeeze-and-Excitation (SE) blocks on multitask learning performance.

Task	Metric	ResNet	ResNet–SE
BIS estimation	MAE	4.08	3.27
BIS estimation	RMSE	6.13	5.48
Deep anesthesia classification	AUC	0.89	0.94
Light anesthesia classification	AUC	0.96	0.99
Transition prediction (3 min)	AUC	0.89	0.94
Transition prediction (5 min)	AUC	0.83	0.91
Transition prediction (10 min)	AUC	0.79	0.85

**Table 8:** Pilot external evaluation results on the NTUH dataset (24 cases, 1901 EEG windows).

Metric	Value
Regression	
MAE	4.69
RMSE	6.63
Classification (AUC)	
Deep anesthesia	0.91
Light anesthesia	0.95
Transition prediction (AUC)	
3 min before BIS60–up	0.82
5 min before BIS60–up	0.81
10 min before BIS60–up	0.78

## Data Availability

The data supporting the findings of this study are openly available in the VitalDB repository at https://vitaldb.net/dataset/ (accessed on 10 March 2025) and the NTUH dataset on Figshare at https://doi.org/10.6084/m9.figshare.5589841 (accessed on 1 June 2025).

## Abbreviations

The following abbreviations are used in this manuscript:

ACC	Accuracy
ASA	American Society of Anesthesiologists
AUC	Area Under the Curve
BIS	Bispectral Index
CNN	Convolutional Neural Network
EEG	Electroencephalography
GAP	Global Average Pooling
IQR	Interquartile Range
LoA	Limits of Agreement
LSTM	Long Short-Term Memory
MAE	Mean Absolute Error
PSD	Power Spectral Density
QC	Quality Control
ResNet	Residual Network
RMSE	Root Mean Square Error
SE	Squeeze-and-Excitation
SHAP	SHapley Additive exPlanations
STFT	Short-Time Fourier Transform
TTE	Time-to-Event
XAI	Explainable Artificial Intelligence
